# Human Milk Oligosaccharides versus Streptococcus: How a Human-Made Natural Product Protects Us from Pathogens

**DOI:** 10.1128/msphere.00049-22

**Published:** 2022-02-16

**Authors:** Laurie M. Lyon, Kelly S. Doran

**Affiliations:** a Department of Immunology and Microbiology, University of Colorado School of Medicine, Aurora, Colorado, USA

**Keywords:** HMOs, group B *streptococcus*, vaginal colonization, vaginal microbiota

## Abstract

Group B Streptococcus (GBS) is a Gram-positive bacterium that colonizes the lower gastrointestinal tract, and in females, the urogenital tract, in up to 30% of healthy adults. However, GBS is a leading cause of mortality and morbidity in newborns due to ascending infection of the womb or by neonatal acquisition during vaginal passage. GBS neonatal disease manifests as pneumonia, sepsis, or meningitis, and an estimated 4 million newborns die each year globally. This commentary reflects on recent work by Mejia and colleagues (M. E. Mejia, S. Ottinger, A. Vrbanac, P. Babu, et al., mSphere 6:e00885-21, 2022, https://doi.org/10.1128/msphere.00885-21) that has examined human milk oligosaccharides (HMOs) as a natural product with anti-GBS activity. They show that HMOs reduce GBS vaginal colonization without impacting the normal vaginal microbiota. This study advances the possibility of using novel therapeutics to limit GBS maternal colonization and subsequent neonatal disease.

## COMMENTARY

## HUMAN MILK OLIGOSACCHARIDES (HMOS) AND THE GUT MICROBIOME

In 1865, the first commercial baby formula, Justus Von Liebig’s Soup for Infants, was introduced with cow’s milk as the primary ingredient (1). As infant formula gained popularity in the late 19th century, it was observed that human milk-fed infants had higher survival and lower rates of infectious diarrheal episodes than formula-fed infants. However, it wasn’t until the mid-20th century that human milk oligosaccharides (HMOs), an indigestible component comprising 8% to 20% of human milk, was discovered to have prebiotic effects in the infant gut (2).

HMOs pleotropic benefits in the infant gut (see recent review by Walsh et al. [3]) include its widely known role as a prebiotic that promotes the growth of beneficial microbes including *Bifidobacteria*. These sugars can also act as decoy lectin receptors at epithelial surfaces to block microbial adhesion, as well as bind dendritic cells to block microbial uptake. HMOs have been shown to help mature the infant gut barrier by increasing tight junction protein expression, ultimately reducing intestinal barrier permeability. Additionally, they function to maintain immune homeostasis by altering cytokine expression through interactions with dendritic and epithelial cells (3). Recent work by Šuligoj et al. proposes that the benefits of HMOs in the gut may extend far beyond infancy. Their study suggests that HMOs can increase *Bifidobacteria* and short-chain fatty acid presence in the gut, decrease paracellular colon cell permeability, and modulate immune function, including decreasing markers of chronic inflammation in adults (4).

## HMOS AND GROUP B STREPTOCOCCUS

While most literature describing HMOs as an antimicrobial agent has focused on the anti-adhesive properties of HMOs against gut microbes, a 2015 study found that HMOs also exhibit a bacteriostatic effect against a prominent maternal-fetal bacterial pathogen, Streptococcus agalactiae, also known as Group B Streptococcus (GBS) (5). GBS is a Gram-positive opportunistic pathogen that commonly colonizes the gastrointestinal and lower reproductive tracts. Ascension of GBS into upper reproductive organs during pregnancy or infection of a neonate during or shortly after birth can result in invasive infections of both the mother and infant. GBS is the leading cause of neonatal sepsis and meningitis in the first week of life (early-onset disease), and can also cause late-onset disease, which manifests 7 days to 3 months after birth (6). Currently, pre-birth vaginal screenings for GBS followed by intrapartum antibiotic prophylaxis is the standard of care in the United States and many developed countries; however, these measures do not significantly protect against late-onset disease and can contribute to the development of antibiotic resistance in GBS and other members of the infant microbiota (6, 7).

In 2016, the first study was published which suggested that mothers producing certain HMOs were significantly less likely to be rectovaginally colonized with GBS at time of labor, and their infants were more likely to clear any GBS colonization within 3 months after birth (8). In the following years, significant work was done to characterize anti-GBS HMO activity *in vitro* (9). Certain HMOs were shown to inhibit growth of GBS independent of host immune interactions (10, 11) and alter GBS biofilm structure (11). Bacteriostatic activity of specific HMOs can also synergize with conventional antibiotics (10) and even re-sensitize certain GBS strains to intracellular-acting antibiotics (12). Additionally, tools have been developed, including synthesized bioorthogonal probes, that are currently being used to identify HMO targets within GBS (13). However, until this year, anti-GBS HMO activity had not been investigated in the context of the vaginal tract. Because GBS is primarily spread to neonates by vertical transmission from vaginally colonized mothers to infants, it is critical to understand how HMOs might act within the vaginal environment against GBS as well as other native vaginal microbes.

## HMOS AS A NOVEL TREATMENT STRATEGY FOR GBS VAGINAL CARRIAGE

In their recent publication, Mejia et al. (14) use an *in vivo* model of murine vaginal colonization to address the impact of HMO treatment on GBS burden and endogenous vaginal microbiota. By coupling the GBS vaginal colonization model with 16S sequencing, the authors found that mice treated with pooled HMOs (pHMOs) before and during infection with GBS had significantly lower GBS burden in the vagina than mock treated groups (14). Importantly, this was achieved without affecting the overall community structure of the vaginal microbiota. This facet of the study was facilitated by the Patras Lab’s prior characterization of discrete murine community state types (mCSTs) within the vaginal tract of this model organism (15).

While HMOs have been found to inhibit some pathogens by disrupting binding to epithelial layers, the authors found that pretreatment with pHMOs did not affect adherence of GBS or probiotic Lactobacillus rhamnosus to human vaginal epithelial cells (hVECs). They further characterized a putative GBS glycosyltransferase previously identified to be responsible for susceptibility to HMOs (10) and found that disruption of this system did not affect GBS *in vitro* adherence or biofilm formation, murine vaginal colonization, or susceptibility to antibiotics and other stressors. Also, they found that concentrations of pHMOs similar to those found in colostrum and human milk inhibited growth of both wild-type GBS and the HMO-resistant mutant, though to a lesser extent (14). The data presented nicely show that anti-GBS activity by HMOs significantly reduced GBS vaginal carriage without compromising the vaginal microbiota, and that the mechanism of HMO-mediated inhibition is distinct from previously published mechanisms observed *in vitro*.

This work represents an important milestone in the study of abundant human-made natural products, such as HMOs, as treatments that can replace or synergize with existing therapies against pathogenic bacteria. Because the vaginal tract, like the gut, is an environment whose health depends on unique microbial communities, we appreciate that this study carefully analyzed the impact on the native vaginal microbiota—a component that is often overlooked in *in vivo* studies of the reproductive tract. It will be interesting to see how the field of anti-GBS HMO activity will evolve in the coming years and how *in vivo* models will continue to explore this promising therapeutic agent for improved maternal and neonatal health.
